# Brown Recluse Spider Bite Mediated Hemolysis: Clinical Features, a Possible Role for Complement Inhibitor Therapy, and Reduced RBC Surface Glycophorin A as a Potential Biomarker of Venom Exposure

**DOI:** 10.1371/journal.pone.0076558

**Published:** 2013-09-27

**Authors:** Eric A. Gehrie, Hui Nian, Pampee P. Young

**Affiliations:** 1 Department of Pathology, Microbiology and Immunology, Vanderbilt University Medical Center, Nashville, Tennessee, United States of America; 2 Department of Biostatistics, Vanderbilt University Medical Center, Nashville, Tennessee, United States of America; 3 Tennessee Valley Veterans Affairs Hospital, Nashville, Tennessee, United States of America; University of Leicester, United Kingdom

## Abstract

**Background:**

The venom of *Loxosceles* *reclusa* (Brown Recluse spider) can cause a severe, life-threatening hemolysis in humans for which no therapy is currently available in the USA beyond supportive measures. Because this hemolysis is uncommon, relatively little is known about its clinical manifestation, diagnosis, or management. Here, we aimed to clarify the clinical details of envenomation, to determine the efficacy of the complement inhibitor eculizumab to prevent the hemolysis *in* *vitro*, and to investigate markers of exposure to Brown Recluse venom.

**Study Design and Methods:**

We performed a 10-year chart review of cases of Brown Recluse spider bite-mediated hemolysis at our institution. We also designed an *in* *vitro* assay to test the efficacy of eculizumab to inhibit hemolysis of venom exposed red blood cells. Finally, we compared levels of CD55, CD59 and glycophorin A on venom exposed versus venom-naïve cells.

**Results:**

Most victims of severe Brown Recluse spider mediated hemolysis at our institution are children and follow an unpredictable clinical course. Brown Recluse spider bite mediated hemolysis is reduced by 79.2% (SD=18.8%) by eculizumab *in* *vitro*. Erythrocyte glycophorin A, but not CD55 or CD59, is reduced after red blood cells are incubated with venom *in* *vitro*.

**Conclusion:**

Taken together, our laboratory data and clinical observations indicate that *L* *. reclusa* venom exposure results in non-specific antibody and complement fixation on red blood cells, resulting in complement mediated hemolysis that is curtailed by the complement inhibitor eculizumab *in* *vitro*. Glycophorin A measurement by flow cytometry may help to identify victims of *L* *. reclusa* envenomation.

## Introduction

*Loxosceles reclusa* ( *L* *. reclusa* ), commonly referred to as the Brown Recluse, is a venomous spider endemic to the southeastern and Midwestern United States [1-5]. The active component of *L* *. reclusa* venom is a broadly reactive, 305 amino acid enzyme [6,7]. Envenomation can result in disfiguring dermatonecrosis and/or a fatal hemolytic anemia [8-11]. The severity of injury has led to the consideration of a number of possible treatments such as dapsone, glucocorticoids and hyperbaric oxygen; but, none of these has been effective and severely ill patients generally receive only supportive therapy [2,8,9]. Although an ELISA for *L* *. reclusa* venom exposure has been described, it is not available for general use [12,13]. There are relatively few case reports and recommendations for the management of Brown Recluse spider bite (BRSB) mediated hemolysis [9,14,15].

Our center, located in the endemic region of *L* *. reclusa* , encounters several cases of severe envenomation per year. The victims we treat are frequently children who follow an unpredictable clinical course requiring inpatient management. In our experience, once BRSB mediated hemolysis becomes clinically apparent it can accelerate rapidly and can be difficult to manage in the absence of a diagnostic test and definitive treatment. Here, we report the salient clinical and laboratory features of 17 cases of severe BRSB mediated hemolysis – the majority of which were in children – as determined by a 10-year retrospective chart review.

Previous investigators have shown that BRSB mediated hemolysis is complement dependent [6,16-19]. However, until eculizumab was approved by the United States Food and Drug Administration (FDA) in 2007, there was no viable complement inhibitor available for clinical use [16,20]. Eculizumab carries FDA labels for the treatment of paroxysmal nocturnal hemoglobinuria (PNH) and atypical hemolytic uremic syndrome (aHUS) and has been used off-label to successfully treat other complement-mediated hemolytic processes such as refractory cold agglutinin disease and catastrophic antiphospholipid antibody syndrome in the setting of renal transplantation [21-23]. Here, we investigate whether eculizumab could curtail BRSB associated hemolysis in an *in vitro* model.

Previous investigators have also determined that levels of a highly expressed RBC membrane protein, glycophorin A, may be altered by exposure to the venom of a different Loxosceles species [17]. In order to clarify the mechanism of BRSB mediated hemolysis, and to move towards the goal of developing an easily accessible laboratory test for *L* *. reclusa* venom exposure, we determined the effect of venom exposure on glycophorin A as well as two membrane bound complement regulatory proteins (CD 55 and CD 59) which are associated with PNH and drug induced immune hemolytic anemia.

## Materials and Methods

### Ethics Statement

This project did not involve interaction with human subjects. Only de-identified specimens and de-identified medical records were used. The IRB at Vanderbilt University Medical Center specifically waived informed consent for use of both adult and pediatric electronic medical records in the Synthetic Derivative research repository, which was used for the chart review. In addition, the chart review on the de-identified records was determined by the Vanderbilt IRB to be a non-human subjects study (Vanderbilt IRB#120695). The specimens used for the laboratory experiments were de-identified and determined to be non-human subjects by the Vanderbilt IRB (Vanderbilt IRB# 110847).

### Chart Review

The 10-year Retrospective Chart Review was conducted within the Vanderbilt Synthetic Derivative version 2.0, a de-identified version of the hospital electronic medical record system that is described in detail elsewhere [24]. Cases were identified by searching for keywords related to Brown Recluse spider envenomation (systemic loxoscelism, loxoscelism, or brown recluse) in the clinical notes of the de-identified medical record. The keyword search produced 399 de-identified medical records in the past 10 years, which were then individually examined by one of the study authors. In order to be considered a confirmed case of BRSB hemolysis, cases had to have clinical documentation of a recent insect bite, signs (hematuria, fever, or jaundice) or symptoms (abdominal pain) supportive of hemolysis, as well as a hematocrit of less than or equal to 20% and an abnormal LDH and/or serum total bilirubin. Seventeen (4%) of 399 patients hospitalized with a confirmed diagnosis of brown recluse spider bite mediated hemolysis were identified. The laboratory and clinical data from each of these 17 cases were recorded.

### Erythrocytes and Plasma

Residual human erythrocytes and fresh frozen plasma from anonymous volunteer blood donors were obtained from the Vanderbilt University, Blood Bank inventory.

### 96-hour Hemolysis Assay

A 96-hour assay was chosen due to the clinical observation that several days typically elapse between envenomation and clinical signs or symptoms of brisk hemolysis. *L* *. reclusa* spider venom was obtained via electrical stimulation (SpiderPharm, Yarnell, AZ) and stored frozen at -80°C until use. Packed erythrocytes were removed from tubing, washed 3 times in 0.9% NaCl, and, after centrifugation, were incubated at 37°C for 30 minutes with thawed spider venom in a ratio of approximately 1 mcg of spider venom protein per mcL of packed RBCs. After incubation, the cells were washed once with PBS and divided into 5 mcL aliquots. Some aliquots were treated with 10 mcL (10 mg/mL; 100 mcg total) of eculizumab, while others were treated with 10 mcL of PBS. Subsequently, the RBCs were immediately suspended with 400 mcL of ABO-identical fresh or heat inactivated plasma (as a negative control) and then incubated in a 37°C water bath for 96 hours. Therefore, in experiments were eculizumab was added, it remained in the assay for the duration of the incubation. After 4, 24, 48, 72 and 96 hours of incubation, the samples were centrifuged and 50 mcL of plasma were removed. After 96 hours, all supernatants were diluted 1:10 with PBS and assayed for hemolysis via spectrophotometry at 405 nm as previously described [7].

### 72-hour Hemolysis, IgG and Complement Deposition Assays

Erythrocytes from donors were washed, exposed to venom, and placed in ABO identical plasma from volunteer donors as described above. After 72 hours of incubation, erythrocytes were assayed for IgG deposition using the ID-Micro Typing System (Micro Typing Systems, FL) and C3 deposition via tube hemagglutination using C3b/C3d antibody (Immucor, Norcross, GA) according to the manufacturer’s guidelines.

### Flow Cytometry

After exposure to Brown Recluse spider toxin or PBS for 30 minutes at 37°C as described above, cells were washed and treated with fluorescent dye conjugated monoclonal antibodies directed against glycophorin A (BD Biosciences, Sparks, MD), CD 55 and CD 59 (eBiosciences, San Diego, CA), similar to previous [25]. Specimens were vigorously pipetted to prevent agglutination. Analysis occurred on an LSR II flow cytometer (BD Biosciences, Sparks, MD) using the FACS Diva software (BD Biosciences, Sparks, MD) and FlowJo software (Treestar, Ashland, OR).

### Statistical Analysis

A linear mixed-effects model was built to examine the impact of different treatments on hemolysis, with linear and quadratic terms of time as well as the interaction between time and treatment as fixed effects and with random effects of experiment and time. Linear mixed-effects regression models were also fitted for glycophorin A, CD 55 and CD 59 to examine the association between expression of these markers and exposure to spider venom with random experiment effect included. Inferences on fixed effects of interest were made by Wald statistics. All analyses were conducted using the R2.10.1 statistical package (http://www.r-project.org) or SAS release 9.3.1 (Cary, NC). P values less than 0.05 were considered statistically significant.

## Results

### Clinical Features of Severe BRSB Hemolysis

**Figure 1:**
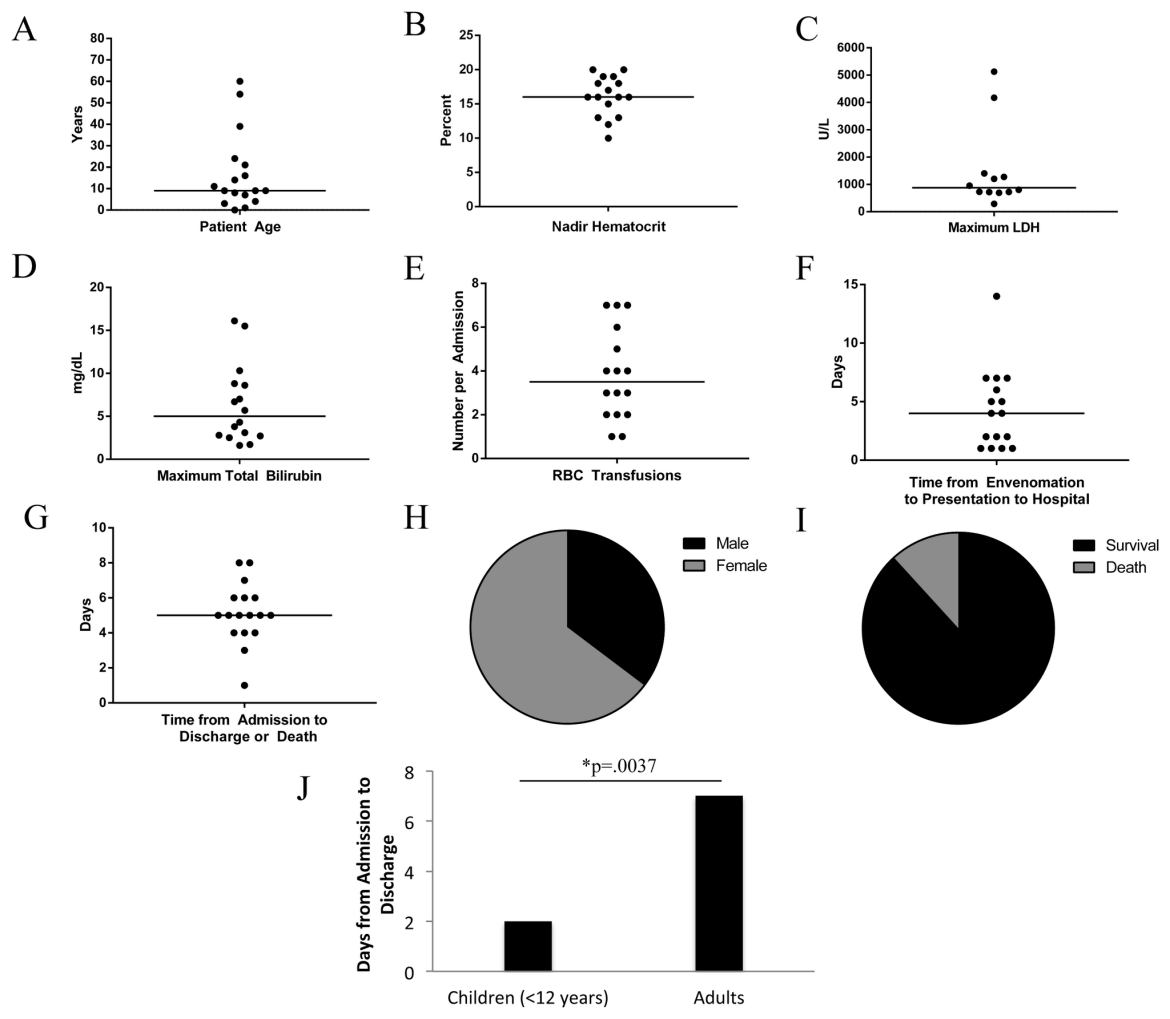
Characteristics of victims of severe Brown Recluse spider bite mediated hemolysis from 2002 through 2012 at Vanderbilt University Medical Center. Horizontal line identifies the median value.

Our 10-year retrospective chart review identified 17 cases of life-threatening BRSB mediated hemolysis at our institution over the past 10 years. The laboratory findings in each of the 17 cases are summarized in Figure 1 and listed in Table S1. The median age of patients was 9 years, and the majority (71%) of cases were age 18 or younger (Figure 1A). Envenomation was associated with a decreased hematocrit (median nadir value = 16%; adult reference range = 42-50%), elevated LDH (median maximum value = 878 U/L; adult reference range = <225 U/L), elevated total bilirubin (median maximum value = 5 mg/dL; adult reference range 0.2-1.2 mg/dL) and a median of 3.5 blood transfusions (Figure 1B-E). On average, patients presented approximately 4 days after envenomation and were admitted to the hospital for 5 days (Figure 1 F-G). Most victims were female (Figure 1H). Two patients died despite supportive therapy, including one toddler (Figure 1I). The case fatalities were ages 4 and 54, were Caucasian versus African American, were male and female, with neck and torso bites, respectively. Typically, young children (under age 12) presented with symptoms sooner after envenomation compared to older patients (median of 2 vs. 7 days until presentation, P =.0037; Figure 1J). All patients were otherwise healthy and had no evident co-morbidities predisposing them to hemolysis. Patients presented with a wound at the site of envenomation, at least one sign of (e.g., hemoglobinuria or jaundice) or symptom (e.g., abdominal pain) of hemolysis.

The direct antiglobulin test (DAT) was not consistently positive for either C3 or IgG on the samples for which data were available, although a previous report demonstrated consistent deposition of complement in envenomed adolescents [9]. In our study, patients who did have a positive DAT for IgG were found to have a negative eluate when tested with panel cells (n=2), indicating that the IgG attached to RBCs *in vivo* did not demonstrate reactivity with common RBC antigens.

### Venom Exposure Results in IgG and Complement Fixation to the RBC surface, resulting in Complement Mediated Hemolysis

**Figure 2:**
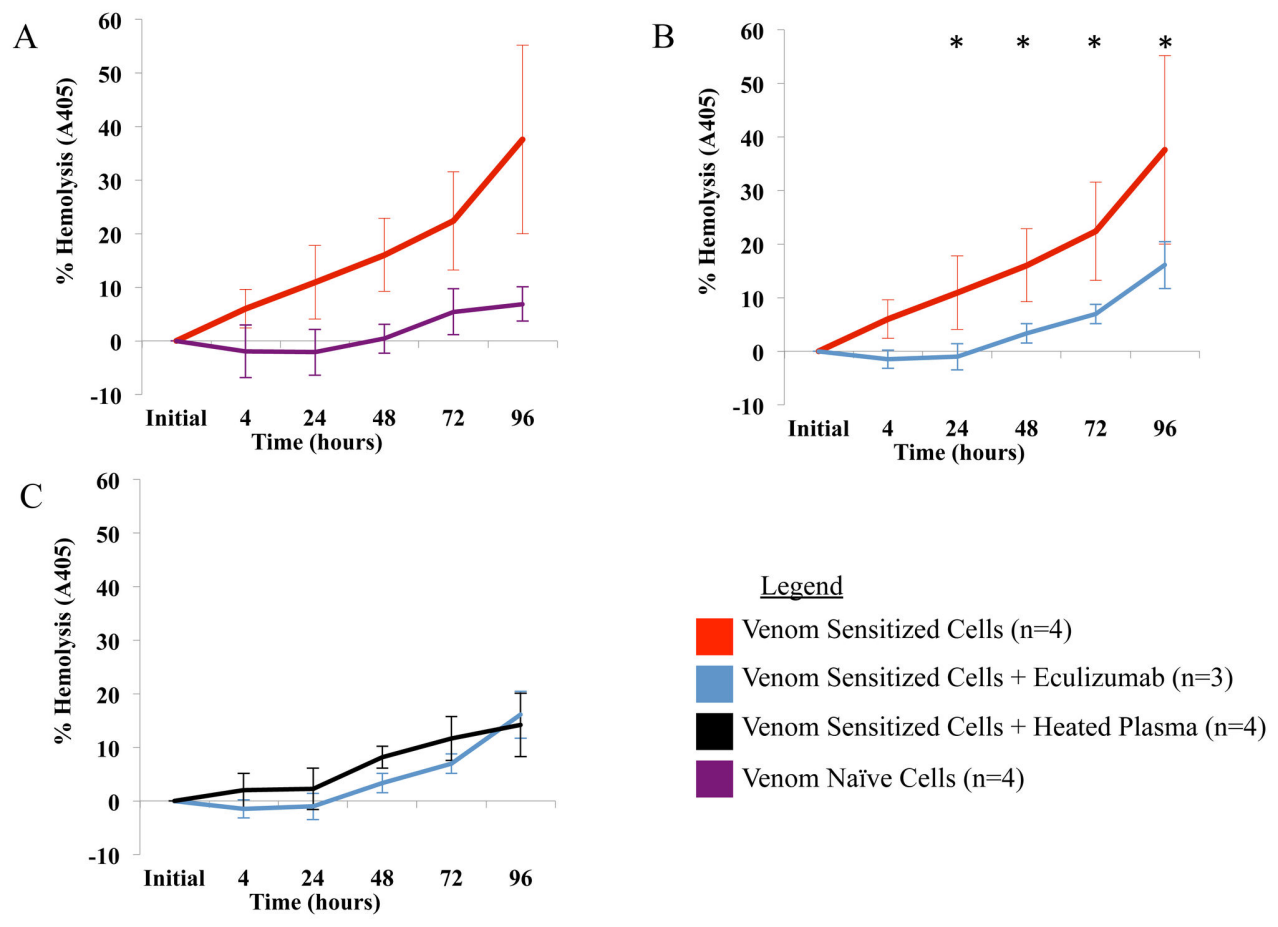
Effect of eculizumab on *L* *. reclusa* associated hemolysis *in vitro*. (A) *L* *. reclusa* venom sensitized cells are hemolyzed over time when incubated with ABO identical plasma, compared to venom naïve RBCs. (B) This hemolysis is reduced by an average of 79.2% (SD = 18.8%) when eculizumab is added to the assay. (C) Eculizumab was statistically indistinguishable from heat inactivation at all timepoints. Results are averages of 3-4 experiments. Error bar shows results +/- 1 standard deviation. Asterisk indicates statistical significance, p<.001.

We found that venom naïve erythrocytes had no detectable IgG or complement bound to surface membranes. However, venom sensitization resulted in deposition of both IgG and complement on the RBC membrane after incubation with ABO-identical fresh frozen plasma (Table 1). In addition, venom sensitized erythrocytes - but not venom naïve erythrocytes - hemolyzed in the presence of ABO-identical fresh frozen plasma (Figure 2A). When complement-poor (heat inactivated) plasma was substituted for fresh frozen plasma from the same donor, hemolysis was almost completely prevented (Figure 2C).

**Table 1:** Venom exposure and incubation with a source of IgG and complement (fresh frozen plasma) leads to IgG and complement deposition on RBCs that initially were negative for complement and IgG.

Donor	**IgG**		**Complement**	
	Untreated	Venom Treated	Untreated	Venom Treated*
1	0	4+	0	1+/4+
2	0	2+	0	w+/2+
3	0	2+	0	1+/2+
4	0	3+	0	0/3+
5	0	3+	0	0/3+

Although venom exposed, plasma incubated washed cells from some donors demonstrates mild autoagglutination, exposure to complement antibody increased the strength of agglutination in all instances. Grading is on a scale of 0 (no agglutination) to 4+ (strong agglutination). w = weak.

* Pre/Postincubation with anti-C3b/d.

### Eculizumab Inhibits *L. Reclusa*-Mediated Hemolysis

**Figure 3:**
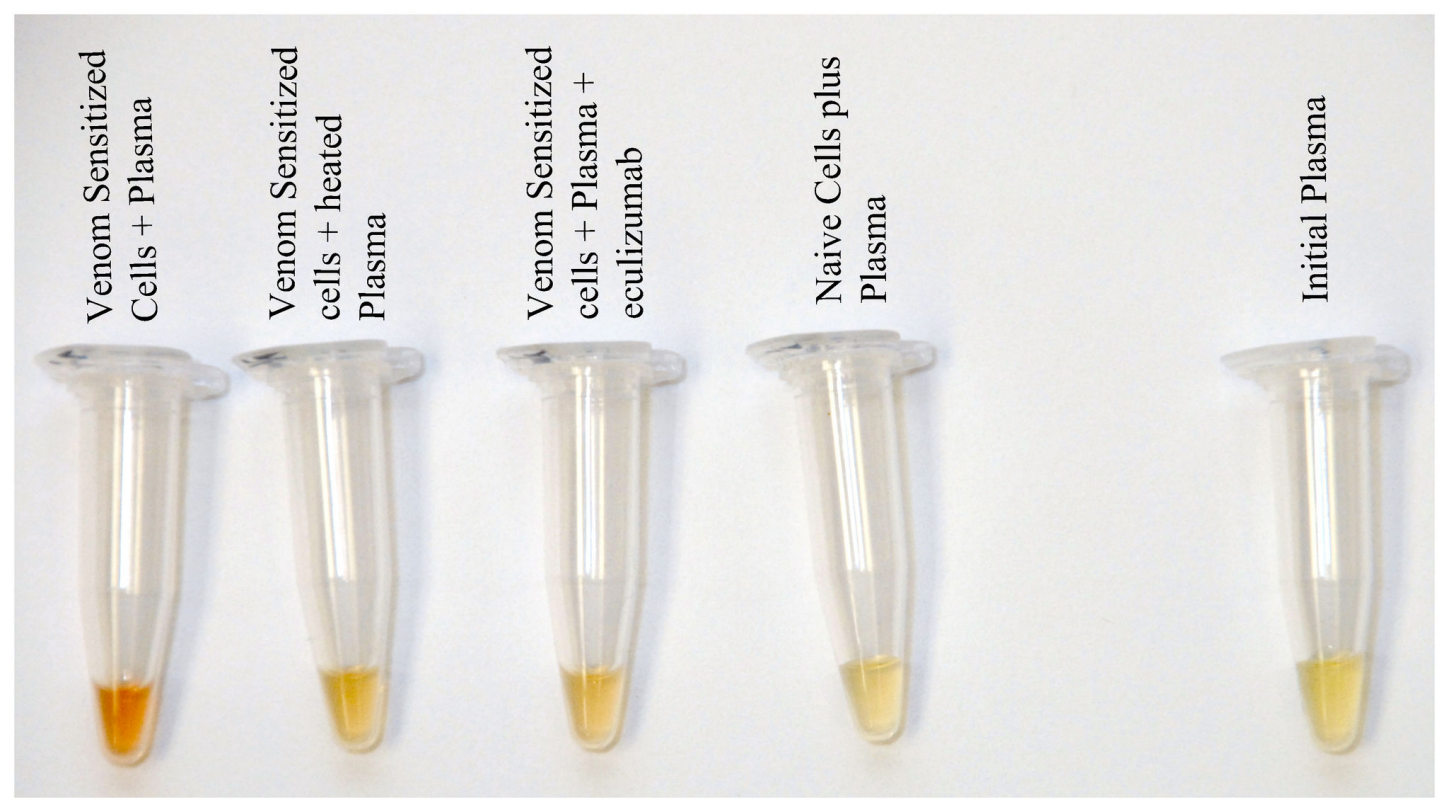
Photographs of plasma under various experimental conditions after 72 hours of incubation, compared to initial plasma (right).

The addition of eculizumab to experiments containing venom exposed RBCs and ABO-identical fresh frozen plasma reduced hemolysis at 24 hours of incubation, from 10.9% hemolysis without eculizumab to none detectable with eculizumab (p<.001). At 48 hours, there was 16.0% hemolysis without eculizumab vs. 3.3% with eculizumab (p<.001). At 72 hours, there was 22.4% hemolysis without eculizumab vs. 6.9% with eculizumab (p<.001). At 96 hours, there was 37.6% without eculizumab vs. 16.1% with eculizumab (p<0.001; see Figure 2B and Figure 3). Eculizumab did not reduce hemolysis to a statistically significant degree 4 hours after exposure: 6% hemolysis without eculizumab vs. none detectable with eculizumab. On average at all time points, eculizumab decreased venom-mediated hemolysis by 79.2% (SD 18.8%). The extent of hemolysis in experiments containing eculizumab was statistically indistinguishable from negative controls consisting of complement-poor (heat inactivated) plasma (p>.05 at all time points, see Figure 2C).

### Venom Exposure Results in Reduced RBC Glycophorin A Expression

**Figure 4:**
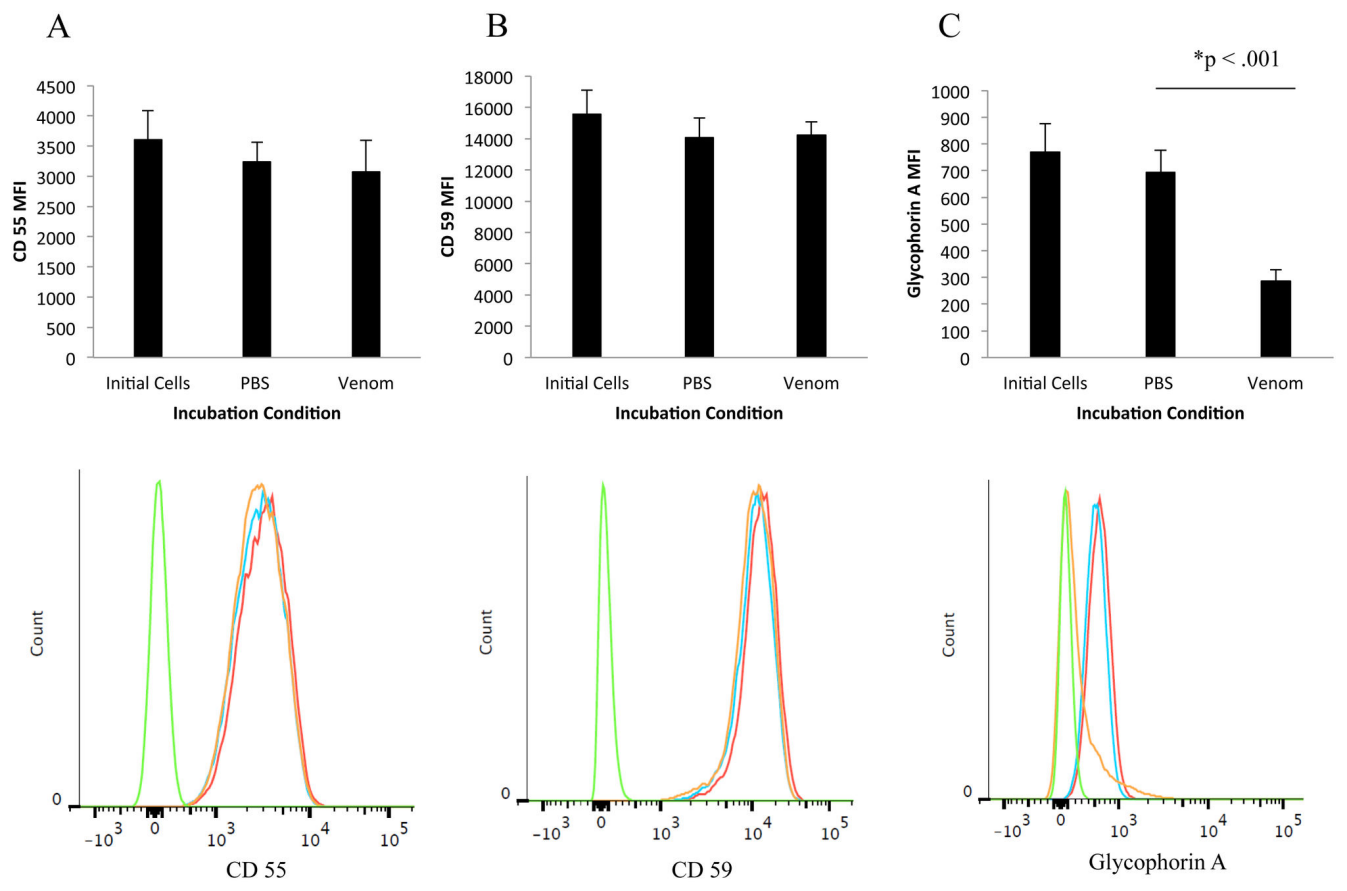
Top Row: Mean data from 5 experiments show that venom exposure results in a statistically insignificant alteration in the expression of CD 55 (A) and CD 59 (B), but a statistically significant reduction of glycophorin A (C). Error bars indicate +/- 1 standard deviation. Bottom Row: Representative histograms from a single experiment (Red = untreated cells; Blue = PBS treated cells; Orange = venom treated cells; Green = isotype control).

Flow cytometry was employed to compare the levels of glycophorin A, CD55 and CD59 in venom naïve versus venom sensitized cells. These experiments showed that exposure to *L* *. reclusa* venom does not significantly change erythrocyte expression of CD 55 or CD 59, compared to venom naive (PBS) controls (P=.079 and P=.714, respectively, see Figure 4A and 4B). In contrast, exposure to *L* *. reclusa* venom dramatically reduced the mean fluorescence intensity (MFI) of glycophorin A on the surface of erythrocytes, compared to incubation with PBS (P<0.001, see Figure 4C).

## Discussion

The majority (71%) of patients with severe BRSB mediated hemolysis in our case series were children under age 18. Our experience and the results of our 10-year retrospective chart review indicate that it is not easy to reliably identify which children (or adults) will develop profound hemolysis (vs. mild to no hemolysis) in the immediate aftermath of *L* *. reclusa* envenomation. This is due the fact that there is no commercially available laboratory test for *L* *. reclusa* venom exposure, nor are there known parameters which will predict whether or not venom-induced hemolysis is likely to occur after envenomation [12,13].

Because transient periods of profound anemia are associated with serious complications such as ischemic stroke [26] and patients may die despite supportive measures, eculizumab (or other systemically active inhibitors of the complement pathway) may have a role in management of severe cases of BRSB mediated hemolysis. The efficacy of eculizumab, which reduced hemolysis by an average of 79.2% at the time points evaluated in our *in vitro* model, establishes a rational basis for the further study of complement inhibitor therapy for the treatment of severe BRSB mediated hemolysis. The risks of profound anemia and exposure to multiple blood donors should be weighed against the possibility of complications – particularly meningitis due to *Neisseria* *meningitis* – which has been reported to result from treatment with eculizumab [21]. We note that recent changes to pediatric meningococcal vaccination schedules [27] indicate that children who are over age 11 should be vaccinated against *Neisseria* *meningitidis* , which may reduce the likelihood of an adverse outcome after eculizumab therapy for a large proportion of victims of envenomation. However, eculizumab does not carry an FDA approval for the treatment of BRSB mediated hemolysis and the clinical utility of eculizumab therapy in this population has not been established *in vivo*. It may also be useful to compare the efficacy of eculizumab against loxosceles anti-venoms, which are not currently FDA approved or available in the United States [28].

Towards the goal of developing a test that could determine whether a patient with an insect bite had been exposed to *L* *. reclusa* venom, we compared the expression of three red cell membrane surface proteins - CD 55, CD 59, and Glycophorin A (CD235a) – in venom exposed versus venom naïve human RBCs. We studied CD 55 and CD 59, which are key complement regulatory proteins, because it is known that levels are reduced in paroxysmal nocturnal hemoglobinuria. In addition, CD55 is altered following exposure of RBCs to cephalosporins [29]. We found no significant alterations in CD 55 and CD 59 levels that would make these assays useful in diagnosis or monitoring victims of BRSB (Figure 4). Our results were consistent with previous investigators studying a different species of Loxosceles spiders [17].

We also evaluated glycophorin A, which is a highly glycosylated structure that carries the M and N blood groups and is associated with the Wright B (Wr^b^), Pr, and high incidence En^a^ blood group antigens [30,31]. It is expressed on erythrocytes in the circulation as well as erythroid precursors in the bone marrow, and the average RBC expresses approximately 1 million glycophorin A structures on its surface [32,33]. Similar to previous investigators studying *Loxosceles intermedia* [17], we found that levels of glycophorin A are reduced to a statistically significant extent in *Loxosceles reclusa* venom-exposed erythrocytes compared to venom naïve erythrocytes.

We considered several possible explanations for the observed reduction in erythrocytic glycophorin A following *in vitro* venom exposure. One possibility is that venom exposure leads to the production of an autoantibody associated with glycophorin A. Support for this theory comes from the observation that some patients with BRSB mediated hemolysis demonstrate IgG deposition on their red cells *in vivo* (positive DAT) [9]. Moreover, cases of severe autoimmune hemolytic anemia with a similar clinical presentation as BRSB mediated hemolysis are known to be caused by autoantibodies directed against minor blood group antigens associated with glycophorin A [30,34,35]. However, we believe that the fact that BRSB mediated hemolysis can be modeled *in vitro* rules out an active immune component (such as autoantibody formation) to the hemolysis. In addition, whereas autoantibodies tend to agglutinate normal cells, in our experience antibodies eluted from the RBCs of victims of BRSB mediated hemolysis do not react with normal cells. Given the ubiquitous expression of glycophorin A on RBCs, we would expect the eluate from affected patients to react with all normal cells if these patients were developing glycophorin A specific autoantibodies. Finally, the timing of hemolysis – within a few days after envenomation - seems too short for the generation of an autoantibody.

A second possibility is that *L* *. reclusa* venom directly hemolyzes RBCs. We believe that this hypothesis is very unlikely, given our observation that venom sensitized RBCs fail to hemolyze when incubated with complement depleted plasma *in vitro*.

A third possibility, and the explanation which we favor, is that exposure to venom enzymatically alters the RBC membrane, making it vulnerable to non-specific attachment of immune globulin and complement, eventually resulting in hemolysis. This hypothesis is supported by two observations: 1) the hemolysis can be modeled *in vitro* and thus does not require an active (specific) immune response; 2) the antibodies eluted from the surface of RBCs from affected patients do not demonstrate reactivity with commonly tested, venom naive RBCs.

This third hypothesis would also suggest that reduction in glycophorin A levels is indicative of venom exposure and not directly causative of RBC lysis. A previous study found that RBCs exposed to venom from a species of *Loxosceles* found only in South America also results in the reduction of erythrocyte glycophorins [17,36]. This may indicate that glycophorin A expression may be used to detect exposure to the venom from various *Loxosceles* species.

The fact that so many of the patients in our case study were children may also lend a clue to the factors that may predispose patients to severe hemolysis. It has been previously reported that *L* *. reclusa* venom attachment to red cells *in vitro* is dose dependent [37]. Perhaps the smaller blood volume of children, in combination with a particularly venomous bite in certain situations, leads to a predilection towards severe disease in the pediatric population. Because so many individuals in the endemic region anecdotally report a history of *L* *. reclusa* spider bite, but very few develop severe hemolysis, other factors such as variations in serum complement and immunoglobulin concentrations or as yet undiscovered genetic factors may also play a role in the immune response to envenomation. When we compared the extent of hemolysis between several combinations of donor RBCs and donor plasma, we found no obvious evidence of individual-specific predisposition to BRSB mediated hemolysis (Figure S1). Further investigation is needed to firmly establish the pathogenesis of this phenomenon.

There are several limitations to our study. First of all, the retrospective chart review was limited by the fact that BRSB mediated hemolysis is a clinical diagnosis that cannot be confirmed by a “gold standard” laboratory assay. We had very stringent case definition criteria – the patients had to have laboratory evidence of severe hemolysis, as well as a clinical presentation and history that led the primary treating team of physicians to have a high suspicion for spider bite hemolysis. This strategy was intended to limit the number of “false positives” in our case series but may have led us to underestimate the true prevalence of this disease at our medical center. Along those lines, many of the victims of envenomation were young children who may not have been able to give a reliable history of the envenomation. If an insect bite was not noted on physical exam, and the clinical team did not suspect a spider bite pathogenesis, then the case would have been missed by our chart review. Second of all, our *in vitro* model was designed to test the efficacy of eculizumab, and therefore used doses of spider venom that are much greater than what would be observed clinically. This approach was intended to help to clarify the mechanism underlying this hemolysis, not as a close approximation of *in vivo* proportions. Further studies are needed to address the optimal timing of eculizumab administration after venom exposure. Third of all, it is difficult to definitively establish the significance of the reduction of glycophorin A levels. Although we are not aware of any disease that reduces expression of glycophorin A, it is possible that the reduction that we observed is fairly non-specific in terms of venom effect on RBCs. Therefore, additional work is needed to determine whether venom from harmless spiders also results in a significant reduction in glycophorin A.

Two of the pediatric patients in our case series were not initially recognized as victims of *L* *. reclusa* envenomation. One patient, a 7 year-old girl, presented one day after suffering a spider bite. She was discharged home with a normal direct serum bilirubin, only to present 4 days later with symptoms and laboratory findings consistent with severe BRSB mediated hemolysis. She was hospitalized for 4 days before recovering. A second patient, a 4 year-old girl, was initially sent home from an outside facility after evaluation for an insect bite. Her symptoms worsened, and she presented to our institution the next day. She eventually died as a result of the BRSB mediated hemolysis, despite supportive measures.

At our institution, we now counsel our clinical colleagues to admit patients with suspected BRSB mediated hemolysis and to obtain frequent hematocrit measurements (every 1-6 hours, depending on the patient’s age and ability to tolerate blood draws) to monitor for hemolysis, which can be sudden in onset. We also recommend frequent monitoring of urine for free hemoglobin, which is especially useful for very young children who are unable to tolerate frequent blood draws. RBC transfusions should be considered when the hematocrit drops below 25-30% or when otherwise clinically indicated.

## Conclusion

In conclusion, *L* *. reclusa* envenomation can result in a life threatening hemolytic anemia with mortality despite supportive measures. Our studies indicate that this hemolytic process is complement mediated and is substantially reduced by eculizumab *in vitro*. Because venom from *L* *. reclusa* results in a reduction of detectable glycophorin A, glycophorin A levels should be further investigated as a supportive test for management of *Loxosceles* venom exposure. A clinical trial to establish the utility of glycophorin A expression and eculizumab therapy in the diagnosis and treatment of *L* *. reclusa* -mediated hemolysis is needed.

## Supporting Information

Figure S1 **Various combinations of venom exposed RBCs (5 donors, labeled 1-5) and compatible plasma (5 donors, labeled A-E) were incubated without eculizumab for 72 hours.** The experiment was performed in duplicate (set 1 and set 2), as were the measurements of hemolysis. When components of variants were estimated by statistical analysis (REML estimate), the largest component of variance was random difference between duplicates (30.3% of total variance, 95% confidence interval 4.73%-24.51%).
(TIF)

Table S1 **Characteristics of 17 victims of severe *L* *. reclusa* associated hemolysis at Vanderbilt University Medical Center, 2002-2012.** n/a = Data Not Available. * denotes fatal cases.
(DOC)
