# Supplementary figures and images for: Brown Recluse Spider Bite Mediated Hemolysis: Clinical Features, a Possible Role for Complement Inhibitor Therapy, and Reduced RBC Surface Glycophorin A as a Potential Biomarker of Venom Exposure
Source: PLoS One. 2013 Sep 27;8(9):e76558. doi: 10.1371/journal.pone.0076558 (PMC3785411; doi:10.1371/journal.pone.0076558)

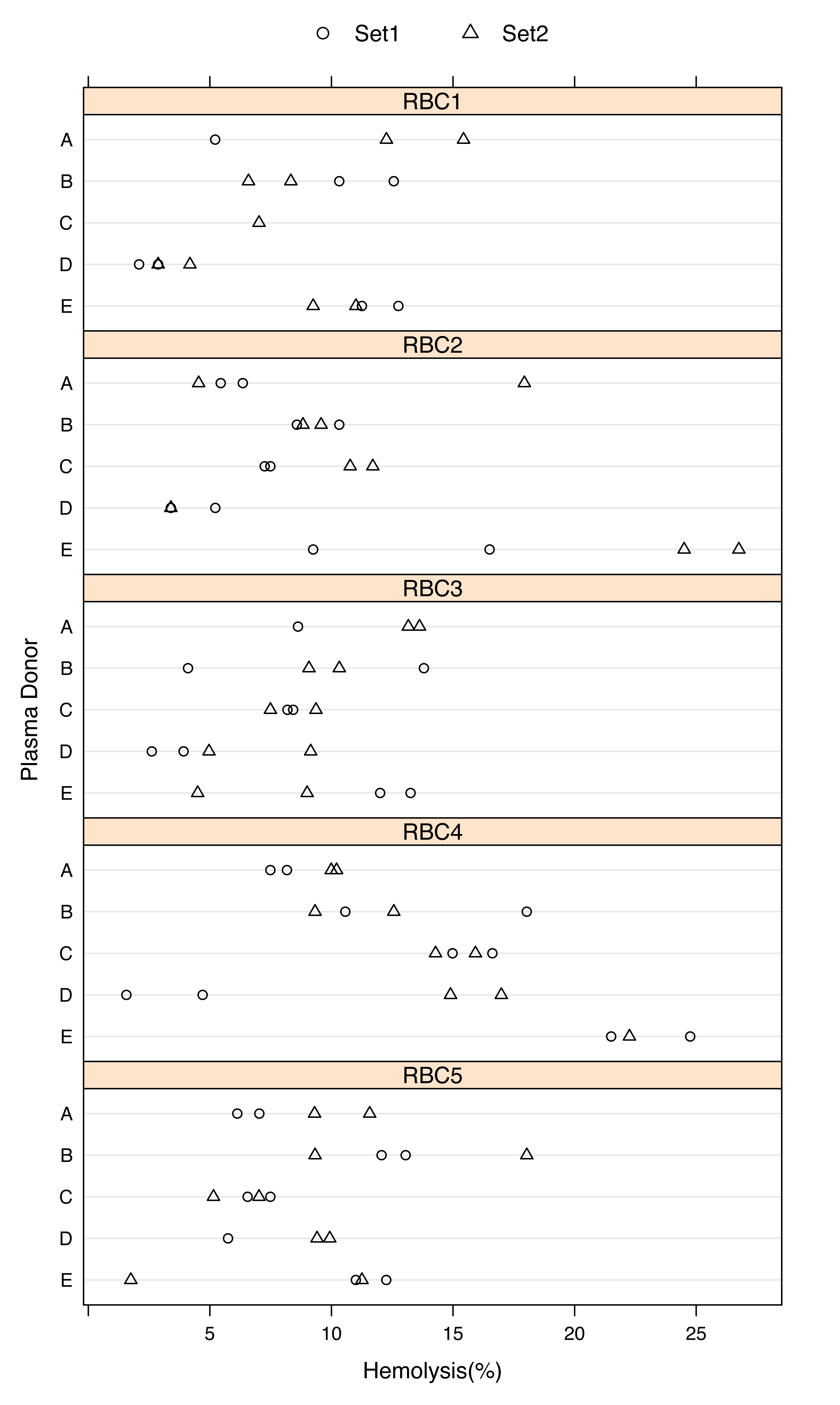

Supplement: Figure S1 — Various combinations of venom exposed RBCs (5 donors, labeled 1-5) and compatible plasma (5 donors, labeled A-E) were incubated without eculizumab for 72 hours. The experiment was performed in duplicate (set 1 and set 2), as were the measurements of hemolysis. When components of variants were estimated by statistical analysis (REML estimate), the largest component of variance was random difference between duplicates (30.3% of total variance, 95% confidence interval 4.73%-24.51%). (TIF) [file pone.0076558.s001.tif]
