# Supplementary material for: Brown Recluse Spider Bite Mediated Hemolysis: Clinical Features, a Possible Role for Complement Inhibitor Therapy, and Reduced RBC Surface Glycophorin A as a Potential Biomarker of Venom Exposure
Source: PLoS One. 2013 Sep 27;8(9):e76558. doi: 10.1371/journal.pone.0076558 (PMC3785411; doi:10.1371/journal.pone.0076558)
Supplement: Table S1 — Characteristics of 17 victims of severe L . reclusa associated hemolysis at Vanderbilt University Medical Center, 2002-2012. n/a = Data Not Available. * denotes fatal cases. (DOC) [file pone.0076558.s002.doc]

| **PMHx** | **Bite Site** | **Bite to Presentation (Days)** | **Hospital Days to Discharge or Death (Days)** | **Presentation** | **Nadir PCV (%)** | **Urine Bilirubin** | **Urine blood** | **DAT IgG** | **DAT C3** | **Max Total Bilirubin (mg/dL)** | **LDH (U/L)** | **RBC Transfusions** | **Hematuria** | **Race** | **Sex** | **Age (Years)** | **Patient** |
| --- | --- | --- | --- | --- | --- | --- | --- | --- | --- | --- | --- | --- | --- | --- | --- | --- | --- |
| Obesity | Thigh | 4 | 5 | Rash, fever, necrosis | 18 | Positive | Large | n/a | n/a | 1.7 | n/a | 3 | Yes | White | F | 16 | **1** |
| None | Torso | 1 | 8 | Hypotension, tachycardia, fever | 17 | Positive | Large | n/a | n/a | 7 | n/a | 3 | No | African American | F | 9 | **2** |
| Asthma | Torso | 7 | 6 | Altered mentation, tachycardia, tachypnea, nausea, vomiting | 16 | Negative | Moderate | Negative | Negative | 3.8 | 719 | 4 | No | Unknown | F | 21 | **3** |
| None | Torso | 1 | 8 | Abdominal pain, headache, wound | 16 | Negative | Negative | n/a | n/a | 1.6 | 285 | 1 | No | White | M | 8 | **4** |
| None | Torso | 1 | 5 | Nausea, vomiting, back pain, fever, jaundice | 19 | Negative | Large | n/a | n/a | 16.1 | 1402 | 2 | Yes | White | F | 3 | **5** |
| None | Axilla | 7 | 5 | Jaundice pruritis, dypnea | 12 | Negative | Moderage | Positive | n/a | 8.8 | 727 | 5 | Yes | White | M | 60 | **6** |
| ADHD | Face | 2 | 7 | Wound, renal failure, dark urine | 18 | n/a | Large | Negative | Negative | 2.8 | 5128 | 7 | Yes | White | F | 9 | **7** |
| None | Axilla | 1 | 6 | Pain, erythema, hematuria | 19 | Negative | Large | Negative | Negative | 3.1 | n/a | 1 | Yes | White | M | 11 | **8** |
| None | Abdomen | n/a | 5 | Rash, hematuria | 20 | Negative | Large | Negative | Negative | 5.7 | n/a | 2 | Yes | White | F | 1 | **9** |
| Hypertension | Torso | 6 | 4 | Weakness, orthostatic hypotension, nausea, vomiting, hematuria | 20 | Negative | Large | Positive | Positive | 8.6 | 1200 | 7 | Yes | African American | F | 39 | **10** |
| None | Shoulder | 5 | 4 | Bite pain, stomach pain, nausea, jaundice, hematuria | 16 | Negative | Trace | Positive | Positive | 6.7 | 954 | n/a | No | African American | F | 7 | **11** |
| Prematurity | Chest | 2 | 4 | Bite pain, hematuria, scleral icterus | 15 | Negative | Large | n/a | n/a | 2.7 | 4170 | 2 | Yes | White | M | <1 | **12** |
| None | Thigh | 7 | 3 | Jaundice, dizzyness, hematuria | 16 | Negative | Trace | Negative | Negative | 10.3 | 690 | 4 | Yes | White | F | 14 | **13** |
| None | Neck | 4 | 5 | Pain, rash, fever, hematuria, hypotension, tachycardia | 13 | Positive | Large | Positive | Positive | 15.5 | 802 | 7 | Yes | African American | M | 54 | **14*** |
| None | Groin | 5 | 6 | Fever, rash | 13 | n/a | Moderate | n/a | n/a | 2.5 | 723 | 4 | No | African American | M | 9 | **15** |
| None | Shoulder | 14 | 5 | Bite pain, fever, abdominal pain, dysuria | 10 | Positive | Large | Positive | Positive | 4.3 | 1270 | 3 | Yes | White | F | 24 | **16** |
| None | Breast | 2 | 1 | Fever, myalgias, hematuria | <limit | n/a | n/a | Negative | Negative | n/a | n/a | 6 | n/a | White | F | 4 | **17*** |
|  |  | 4.0 | 5.0 |  | 16.0 |  |  |  |  | 5 | 878 | 3.5 |  |  |  | 9.0 | **Median** |
|  |  | (1,14) | (1,8) |  | (<, 20) |  |  |  |  | (1.7,16.1) | (285,5128) | (0,7) |  |  |  | (<1, 60) | **Range** |
